# A Perspective on the Development of Plant-Made Vaccines in the Fight against Ebola Virus

**DOI:** 10.3389/fimmu.2017.00252

**Published:** 2017-03-10

**Authors:** Sergio Rosales-Mendoza, Ricardo Nieto-Gómez, Carlos Angulo

**Affiliations:** ^1^Laboratorio de Biofarmacéuticos Recombinantes, Facultad de Ciencias Químicas, Universidad Autónoma de San Luis Potosí, San Luis Potosí, San Luis Potosí, Mexico; ^2^Grupo de Inmunología & Vacunología, Centro de Investigaciones Biológicas del Noroeste, SC., La Paz, Baja California Sur, Mexico

**Keywords:** Ebola virus, mucosal immunization, low-cost vaccine, global vaccination, molecular pharming, glycoprotein antigen, VP antigen

## Abstract

The Ebola virus (EBOV) epidemic indicated a great need for prophylactic and therapeutic strategies. The use of plants for the production of biopharmaceuticals is a concept being adopted by the pharmaceutical industry, with an enzyme for human use currently commercialized since 2012 and some plant-based vaccines close to being commercialized. Although plant-based antibodies against EBOV are under clinical evaluation, the development of plant-based vaccines against EBOV essentially remains an unexplored area. The current technologies for the production of plant-based vaccines include stable nuclear expression, transient expression mediated by viral vectors, and chloroplast expression. Specific perspectives on how these technologies can be applied for developing anti-EBOV vaccines are provided, including possibilities for the design of immunogens as well as the potential of the distinct expression modalities to produce the most relevant EBOV antigens in plants considering yields, posttranslational modifications, production time, and downstream processing.

## Introduction

**Figure 1:**
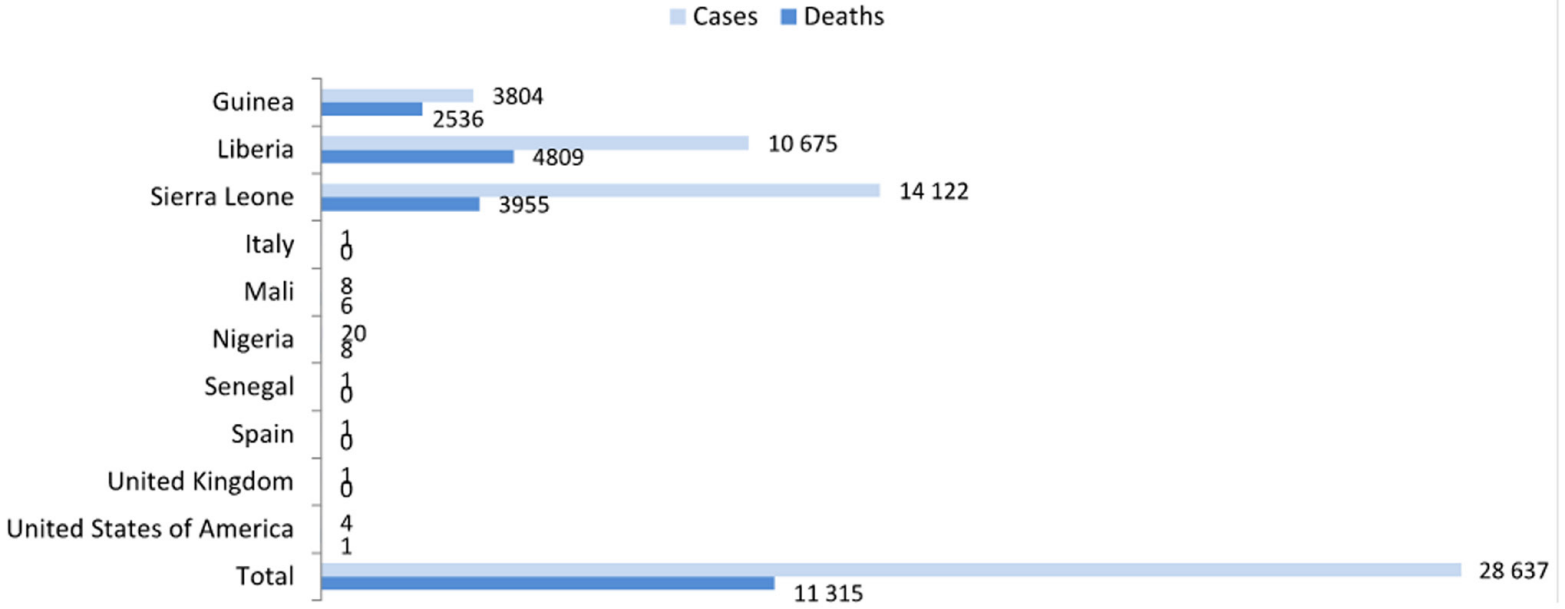
**Confirmed, probable, and suspected EBOV disease cases worldwide (data up to 27 December 2015; report of December 30 from the World Health Organization, http://www.who.int/en/)**.

The last *Zaire Ebola virus* (EBOV) epidemic outbreak in Guinea, which began in December 2013, quickly spread and six West-African countries were greatly affected (Guinea, Liberia, Sierra Leone, Mali, Nigeria, and Senegal). There have also been reports of cases within health-care workers from the USA, Spain, and the United Kingdom. Fortunately, the overall case incidence has dropped, and no reports on confirmed cases during the last week of December 2015 were generated. Nonetheless, according to a report on December 27, 2015, there have been 25,637 confirmed, probable, or suspected cases of EBOV disease (EVD) in Guinea, Liberia, and Sierra Leone (Figure 1), with over 11,000 reported deaths, which surpasses all previous EBOV outbreaks combined (World Health Organization^1^). Therefore, the EBOV constitutes an imminent and serious threat to public health, as well as a potential bioterrorism agent (1). EBOV represents one of the three genera composed of the family Filoviridae (2). The EBOV genus comprises five species: (1) *Sudan ebolavirus* (SUDV), (2) *Zaire ebolavirus* (ZEBOV), (3) *Côte d’Ivoire ebolavirus* (also known as Ivory Coast ebolavirus or Tai Forest ebolavirus, TAFV), (4) *Reston ebolavirus* (RESTV), and (5) *Bundibugyo ebolavirus*. All of these species, with the exception of the RESTV, have shown to cause disease in human beings (3, 4). After an incubation period of 3–21 days, the EVD generally progresses quickly, with symptoms of fever, diarrhea, vomiting, systemic inflammatory response syndrome, organ dysfunction, and hemorrhagic manifestations that end in death (5).

Despite the substantial efforts made to develop rational prophylactic and chemotherapeutic interventions, no licensed countermeasures are available for the treatment of EVD as of now. EBOV is introduced into the human population through close contact with bodily fluids of infected animals such as primates and fruit bats. EBOV then spreads through human-to-human transmission *via* direct contact (through broken skin or mucous membranes) with bodily fluids of infected people. Therefore, most efficient measures to control the EVD spread consist of the isolation of patients establishing strict barrier nursing procedures to protect health-care workers (5). Looking at this situation, the development of effective therapeutics for the prevention and treatment of EBOV infections is urgently needed. In the case of immunotherapies, achieving broad and long-lasting humoral immunity at the mucosa and systemic levels against many EBOV species as possible is a key goal (6). The most advanced immunotherapy against EVD is ZMapp (Mapp Biopharmaceutical, San Diego, CA, USA), a drug consisting of humanized monoclonal antibodies (mAbs) capable of neutralizing the EBOV. This treatment, based on passive immunity, has been successful in non-human primates (NHPs) and efforts for its licensing and introduction into the market are ongoing (7). ZMapp has already been used on a compassionate basis to treat a few patients of EVD; however, the clinical efficacy of this specific cocktail as a treatment of EVD in humans remains uncertain (8). Vaccination is the ideal approach to fight this disease since prophylaxis could be achieved through the administration of a minimum number of doses. Vaccinology offers a myriad of possibilities for the development of vaccines against EBOV, and according to the ClinicalTrials.gov database,^2^ 47 studies of Ebola vaccine trials have been registered. One of the biggest challenges in achieving global vaccination is developing production platforms accessible to developing countries. For instance, protein subunit vaccines are obtained, distributed, and administered through processes requiring complex downstream steps, cold chain, and delivery systems that involve specialized personnel and equipment. All of these aspects hamper vaccination availability and usage in developing countries. Therefore, the next-generation platforms for vaccine production, distribution, and delivery have been proposed to develop low-cost and broad coverage vaccination strategies. In this context, plant-based platforms constitute an attractive technology with the following attributes: (i) since the use of sophisticated bioreactors and complex downstream processing are avoided, the cost of a plant-derived product is 10–50 times lower than products derived from the fermentation with *Escherichia coli* (9) and 140 times lower when compared to baculovirus-infected insect cells (10); (ii) high biosynthetic capacity derived from a machinery that performs folding, assembly, and glycosylation; (iii) the plant systems offer high safety in the sense that they are not hosts of human or animal pathogens, in contrast to mammalian-based production systems where the risk of contamination with viruses and prions exists. Moreover, many plant tissues and fruits are safe for human consumption and thus can be used as oral delivery vehicles for vaccines, thereby avoiding the purification and processing required for conventional injectable vaccines. Therefore, plant-made oral vaccines can be easily formulated with freeze-dried plant material, which not only increases antigen concentration but also produces a material stable at room temperature avoiding the cold chain maintenance required for other delivery systems (11). This perspective constitutes the ideal case for vaccine development, and it has been consolidated in recent years with the successful delivery of many vaccines and other biopharmaceuticals by the oral route in test animals (12–14). The technology of plant-based vaccines and the current advances have been recently reviewed by distinct groups (15–17). During the last years, clinical trials have been conducted to evaluate the immunogenicity and safety of influenza virus vaccines with positive outcomes (18–21), which has stimulated the interest of the pharmaceutical industry in these platforms (Table 1).

**Table 1:** **Evaluations of plant-made vaccines in clinical trials reported over the last years**.

Target disease	Antigen	Expression platform	Outcomes	Reference
Influenza virus, 2009 pandemic A/California/04/2009 (H1N1) strain	Hemagglutinin	Plant virus-based transient expression technology in *Nicotiana benthamiana* plants	Safety and immunogenicity of the plant-produced subunit H1N1 influenza vaccine was proven. No serious adverse effects were observed	Cummings et al. (20)
Influenza virus, A/Indonesia/05/2005 (H5N1) strain	Hemagglutinin	Plant virus-based transient expression technology in *N. benthamiana* plants	Safety and immunogenicity of the plant-produced subunit H5N1 influenza vaccine was proven. No serious adverse effects were observed	Chichester et al. (19)
H1N1 A/California/7/09 (H1) or H5N1 A/Indonesia/5/05 (H5)	Hemagglutinin	Plant virus-based transient expression technology in *N. benthamiana* plants	Besides strong antibody responses, both vaccines elicited significantly greater poly-functional CD4(+) T cell responses	Landry et al. (22)
			H1 vaccine induced poly-functional CD8(+) T cell responses	

In this review, the use of the technology of plant-based vaccines to develop attractive EBOV vaccines is placed in perspective. After describing the molecular approaches to express antigens in the plant cell, the relevant aspects of EBOV as well as conventional vaccines under development were are summarized; finally, the perspectives on how plant systems may lead to EBOV vaccines are identified and discussed.

## Current Experimental Vaccines to Fight EBOV

While the precise mechanisms for immune protection against the EBOV infection are likely complex, it is noteworthy that vaccination against the EBOV surface glycoprotein (GP) is both necessary and sufficient for protection against virus infection as has been evidenced by several successful vaccination approaches (23, 24). This evidence suggests an important role of the GP in virus survival within the host. Several studies have pointed out that the humoral responses induced by these vaccines are strongly associated with protection (25–27), although some reports have clearly demonstrated that the cellular response aided in infection clearance as well (28).

The most advanced vaccines against EBOV are based on the viral GP that has demonstrated protection against EBOV in NHPs. It is important to point out that in 2002, the US Food and Drug Administration introduced the “animal rule” concept that aims to facilitate the licensing of vaccine or drug treatments against infection by the EBOV as well as other highly lethal human pathogens for which the efficacy evaluation in human beings would be unethical and field trials unreasonable (29). The application of the “animal rule” allows for the approval of any EBOV vaccine candidate based on efficacy testing in animal models, with defined immune correlates of protection, as well as Phase I and II clinical trials for safety and immunogenicity testing in human beings. Therefore, the development of animal models is critical for the evaluation and eventual approval of EBOV vaccine candidates. Promising animal models for investigating EBOV vaccines include Guinea pig (30), mouse (31), Syrian Golden hamster (32), marmoset (33), and ferret (34, 35). However, NHP models of EBOV infection, especially the model of cynomolgus macaques, have a stronger predictive value for human diseases and immune protection, and thus, it is the preferred model for EBOV vaccine development (24). Therefore, this review will focus on the most advanced vaccines that have been tested in NHPs and clinical trials. New promising vaccine candidates evaluated using other animal models will be mentioned briefly.

Overall, the candidate vaccines against the EBOV developed thus far can be divided into three categories: non-replicative expressing vector-based vaccines, replication-competent viral vector-based vaccines, and viral antigen-based vaccines (36). Most of the successful vaccines against the EVD rely on viral vectors in whose genome the EBOV GP gene was introduced (37). The vector-based vaccines have been evaluated in NHP and in clinical trials, whose outcomes are summarized in the following sections. Viruses used as vaccine vectors include vesicular stomatitis virus (VSV) (38), recombinant adenovirus replicons (39), recombinant parainfluenza virus (40), recombinant rabies virus (RABV) (41), and recombinant Venezuelan equine encephalitis virus (VEEV) (42). Protein-based vaccines such as virus-like particles (VLPs) have also demonstrated EVD protection in NHPs, but the characterization of most of the candidates has been performed in small animal models (43, 44). A general overview on the progress achieved for each type of vaccine is described in the following sections.

### Non-Replicative Vector-Based Vaccines

Sullivan et al. (45) reported the first proof of concept on protection against EBOV infection by vaccination. The study revealed that priming with an EBOV GP DNA vaccine followed by boosting with a recombinant adenovirus-5 replicon expressing GP conferred complete protection against a lethal EBOV challenge in NHPs. Although promising and safe for human beings, the use of the most advanced adenovirus 5 replicon-based vaccine faces the problem of pre-existing immunity against the viral vector as well as a relatively low immunogenicity in human beings, since the antibody titers against GP were less than 300, while titers of 2,000 are associated with the protection of NHPs (46). An interesting alternative that may solve the problem derived from the pre-existing immunity against the vaccination vector consists in the use of Chimpanzee adenovirus-based vaccines (47).

Another encouraging example is a VEEV replicon that has been employed for EBOV vaccine development. VEEV replicons expressing EBOV GP and *Sudan ebolavirus* (SUDV) GP protected NHPs against a lethal EBOV as well as SUDV challenge when administered a single [1 × 10^10^ focus-forming units (FFU)] simultaneous intramuscular vaccination (42). However, similar to adenovirus replicons, the requirement of high vaccine doses and a pre-existing immunity to VEEV will likely be the major obstacles for human application of this kind of vaccine. A mutant form of the EBOV, without the VP30 gene that is required for virus replication, was evaluated in mice and guinea pigs, and it was shown to confer complete protection against a lethal EBOV challenge after two immunizations (48). Moreover, the efficacy of this new replication-defective viral vector-based vaccine was also confirmed to confer immunoprotection in NHPs when administered by the intraperitoneal route twice at 3-week intervals with 1 × 10^6^ FFU of Ebola ΔVP30 virus. However, this approach raised concerns with respect to virulence reversion, and thus, a new version of the vaccine consisted of the virus inactivated with hydrogen peroxide was generated, which remained antigenic and protective in NHPs when administered intramuscularly (1 × 10^7^ FFU) one or two times with a 4-week interval (49).

Two replication-incompetent vectored vaccines have reached Phase III clinical trials: human adenovirus serotype 26 (Ad26) expressing the Ebola virus Mayinga variant GP (Ad26.ZEBOV) and Modified Vaccinia Virus Ankara-Bavarian Nordic Filo-vector (MVA-BN-Filo). Remarkably, Ad26.ZEBOV and MVA-BN-Filo vaccines resulted in sustained elevation of specific immunity, and no vaccine-related serious adverse events were observed in Phase I clinical trial. In this evaluation, the vaccinated (i.m.) groups were (1) with MVA-BN-Filo as prime vaccine on day 1 boosted by Ad26.ZEBOV on day 29 or day 57; and (2) with a priming dose of Ad26.ZEBOV boosted by MVA-BN-Filo on day 29 or day 57 (50). Therefore, Phases II and III were pursued. Moreover, the Phase IV, named “Long-term Safety Follow-up of Participants Exposed to the Candidate Ebola Vaccines Ad26.ZEBOV and/or MVA-BN-Filo” is active but not open for participant recruitment yet.

### Replication-Competent Viral Vector-Based Vaccines

This category includes rhabdovirus-based viral vectors, including the VSV and RABV, and paramyxovirus-based vectors such as recombinant human parainfluenza virus 3 (HPIV3) expressing EBOV GP separately or in combination with nucleoprotein (NP). The potential of this kind of vaccine platform was shown when the recombinant VSV expressing the GPs of ZEBOV (strain Mayinga) was generated using the infectious clone for the VSV Indiana serotype. A single intramuscular immunization, measured in plaque-forming units (PFU) of the virus particles, of cynomolgus macaques (1 × 10^7^ PFU) demonstrated to protect NHPs against a lethal challenge (1 × 10^3^ PFU) of ZEBOV (strain Kikwit) isolated from a patient from the 1995 EBOV outbreak in Kikwit (38). Similarly, Marzi et al. (51) found complete protection of NHPs against ZEBOV (strain Makona) following the administration of a single dose given as late as 7 days before challenge in VSV–EBOV GP vaccinated animals. Looking to explore practical delivery routes, effective protection of NHPs was observed when the vaccine was administered either orally or intranasally with the subsequent EBOV challenge (52, 53). These findings opened the path to explore mucosal vaccination as a feasible strategy in combating the EVD. Furthermore, this vaccine platform showed potential as an early treatment since it induced beneficial effects in NHPs infected with the EBOV (54) and in individuals who have experienced incidental exposure or high-risk occupational exposure to the EBOV such as a needle stick handling (55, 56). Interestingly, a single intramuscular immunization (1 × 10^7^ PFU in the caudal thigh) of the full-length parent RABV vaccine expressing the EBOV GP also conferred complete protection in rhesus macaques after a challenge with 1,000 PFU of the EBOV (strain Mayinga). However, its potency was lower when compared to recombinant VSV-based vaccines (41), such as the attenuated vesiculovax recombinant VSV-based vaccines expressing the EBOV GP, which protects macaques from a lethal challenge after a single dose (57). Another vaccine platform uses a paramyxovirus-based vector, such as the recombinant HPIV3 expressing EBOV GP alone or in combination with NP. These vaccines were constructed by inserting a transcription cassette encoding the EBOV (Mayinga strain) GP gene between the HPIV3 P and M genes alone or in combination with a cassette encoding the NP inserted between the HPIV3 HN and L genes. Rhesus monkeys were protected against the EBOV infection after receiving two doses of 2 × 10^7^ tissue culture infectious dose (TCID50) (days 0 and 28) of combined intranasal and intratracheal inoculation and an intraperitoneally challenge on day 67 (39 days following the second vaccine dose) with 1,000 PFU of the EBOV (*Zaire* species, Mayinga strain) (40). This study reinforces the practical feasibility of immunization against the EVD *via* the respiratory tract (58). However, since these vaccine platforms are replication competent, their side effects for human vaccination is a major concern and merits further research. Recently, Phase I and II clinical trials have been conducted, and the results showed that rVSV-ZEBOV is immunogenic but also mild to moderate reactogenic. rVSV-ZEBOV used at 1–5 × 10^7^ PFU (Phase I) provoke fever (25%) and oligoarthritis (22%) in vaccinated volunteers (6). A reduced dose of 3 × 10^5^ (Phase II) PFU decreases viremia and reactogenicity but also antibody response levels without reducing the risk of vaccine-induced side effects (59). Remarkably, a Phase III trial in Guinea highlighted that the rVSV-ZEBOV is highly efficacious when administered in a single 2 × 10^7^ PFU dose (estimated vaccine efficacy of 100%) and safe in preventing the EVD, while the assessment of vaccine-derived adverse events revealed promising outcomes (2 serious adverse events in 5,837 vaccinees) (60, 61).

### Viral Protein/DNA-Based Vaccines

Konduru et al. (62) provided the first proof of concept that a subunit vaccine based on purified GP could elicit protective immune responses against the EBOV. In their study, a ZEBOV GP-Fc fusion protein was constructed coding for the C-terminal end (1–637 aa) of the extracellular domain from the ZEBOV GP (Mayinga strain) and the crystallizable fragment (Fc) from human IgG1. The ZEBOV GP-Fc protein fusion was produced in transfected Chinese hamster ovary cells. C57BL/6 mice were intraperitoneally vaccinated (i.p.) with 100 μg of purified ZEBOVGP-Fc in complete Freund’s adjuvant and boosts (25 μg in incomplete Freund’s adjuvant) were administered at 21, 45, and 60 days post-priming. A 90% of protection in the vaccinated mice was achieved after a lethal challenge by i.p. injection with 1,000 PFU of mouse-adapted ZEBOV. Similar results were obtained by Phoolcharoen et al. (63) in which the GP was fused to a mAb that recognizes an epitope in the GP, resulting in the production of EBOV immune complexes (EICs). Remarkably, the EICs were produced in *Nicotiana benthamiana* plants by transient expression. The purified EICs were tested in mice, administered by the subcutaneous route four times on days 0, 21, 42, and 63, and the immunogenic properties determined. Although antigen–antibody immune complexes were efficiently processed and presented to immune effector cells, they found that co-delivery of the EIC with toll-like receptor (TLR) agonists elicited a more robust antibody response in mice than the EICs alone. Among the compounds tested, polyinosinic:polycytidylic acid (poly I:C, a TLR-3 agonist) was highly effective as an adjuvant. After vaccinating mice with EIC plus poly I:C, 80% of the animals were protected against a lethal challenge with live EBOV. These results are encouraging but further research is needed to optimize the immunogenicity of this vaccine and test its efficacy in NHP models with the subsequent determination of its safety in clinical trials. Another viral antigen-based vaccine strategy is the use of VLP that can direct the target antigen to antigen presenting cells, such as dendritic cells, stimulating antibody, and cellular immune responses. Interestingly, three i.m. immunizations at 42-day intervals with enveloped EBOV VLPs containing the EBOV GP, NP, and VP40 matrix protein, along with RIBI adjuvant, conferred protection to NHPs against a lethal challenge with EBOV, thus providing the first evidence that protective immunity can be elicited by non-viral vector-based vaccines in NHPs (43). Moreover, the versatility of VLPs should be noted that they can either be used as carriers of immune-stimulating molecules or enriched with chimeric EBOV GP carrying additional epitopes as an approach to enhance immune responses.

It is also important to note that the results on three DNA vaccines (INO-4201, -4202, and -4212) and one recombinant protein subunit vaccine (EBOV GP1,2 with Matrix-M) have not been published yet and probably will bring new perspectives in the race of developing new Ebola vaccines (64). DNA vaccines expressing the EBOV GP have also been tested in human beings during Phase I clinical trials with safe and immunogenic properties when applied under a scheme comprising three i.m. doses (2, 4, and 8 mg) on days 0, 28, and 56 (65) and an homologous boost (2 mg) at week 32 or after (66).

## Perspectives for EBOV Vaccine Development

Despite the milestone of establishing durable protection against the EBOV, future developments are required to increase qualitative or quantitative resolution of the protective and non-protective humoral immune responses (67). Two encouraging vaccines based on GP have been evaluated under Phase I and Phase II clinical trials (Table 2) showing durable protection in the cynomolgus macaque model (47, 68, 69). Based on promising data from the initial clinical trials, gathered in the late 2014, the WHO in combination with the Health Ministry of Guinea, Médecins Sans Frontières from Epicentre, and The Norwegian Institute of Public Health launched a Phase III trial in Guinea on March 7, 2015. This trial tested the VSV–EBOV (VSVΔG-ZEBOV-GP) vaccine for efficacy and effectiveness in preventing the EVD (60). The results indicated that the vaccine is highly efficacious and safe, and likely effective in the population when delivered during an EVD outbreak *via* a ring vaccination strategy (60). In addition, the plan includes testing another advanced vaccine called ChAd3 (ChAd3-ZEBOV-GP; GSK). The follow-up study to compare the safety and efficacy of the ChAd3 Ebola *Zaire* and VSVΔG-ZEBOV-GP virus vaccines through Phase II/III clinical trials in volunteers from Liberia led to promising results upon the first 4 months, and serious adverse effects were not reported (24, 64, 70). ChAd3 is an example that the current EBOV vaccines require cell-based production and storage at low temperature, thereby creating obstacles in scalable manufacturing and shelf-life in developing countries (67).

**Table 2:** **Current EBOV Food and Drug Administration-approved vaccine trials**.^a^

Vaccine platform	Trial type	Start date^b^	Location	Enrollment^c^	Sponsor
Chimpanzee adenovirus vector (ChAd3-ZEBOV-GP)	Phase I a/b dose escalating	2014 August	USA (Georgia and Maryland)	26	National Institute of Allergy and Infectious Diseases, USA
	Phase Ia dose escalating	2014 September	United Kingdom	60	University of Oxford, UK
	Phase I/II	2014 October	Lausanne, Switzerland	120	University of Lausanne Hospitals, Switzerland
	Phase Ib dose escalating	2014 November	Mali, Africa	40	University of Maryland, USA
Vesicular stomatitis virus vector (VSVDG-ZEBOV-GP) (37, 53)	Phase Ia dose escalating	2014 August	USA (National Institutes of Health, Maryland)	120	NewLink Genetics, USA
	Phase Ia dose escalating	2014 October	USA (Walter Reed Army Institute of Research, Maryland)	117	NewLink Genetics, USA
	Phase I/II	2014 November	Geneva, Switzerland	115	University Hospital, Geneva, Switzerland
	Phase I	2014 November	Germany	30	Hamburg-Eppendorf, Germany
Human adenovirus serotype 26 (Ad26) expressing the Ebola virus Mayinga variant glycoprotein (GP) (Ad26.ZEBOV) and Modified Vaccinia Virus Ankara-Bavarian Nordic Filo-vector (MVA-BN Filo), in a heterologous prime-boost regimen	Phase III	2015 September	Kambia, Sierra Leone	This study is currently recruiting participants	Crucell Holland BV

*^a^Information was collected from public records provided by the U.S. National Institutes of Health and is current as of March 2016 (https://clinicaltrials.gov/ct2/home)*.

*^b,c^May represent proposed dates and enrollments, respectively*.

Overall, non-replicative vector-based vaccines face the problem of pre-existing immunity and/or the induction of anti-vector immune responses that may decrease their efficacy, while viral replication-competent vaccines face important human safety or adverse side effects concerns. By contrast, the vaccine strategies based on viral protein antigens are not affected by those issues. In this context, plant-made vaccines can be a reasonable alternative in the fight against the EVD.

## How Could EBOV Plant-Based Vaccines be Developed?

**Figure 2:**
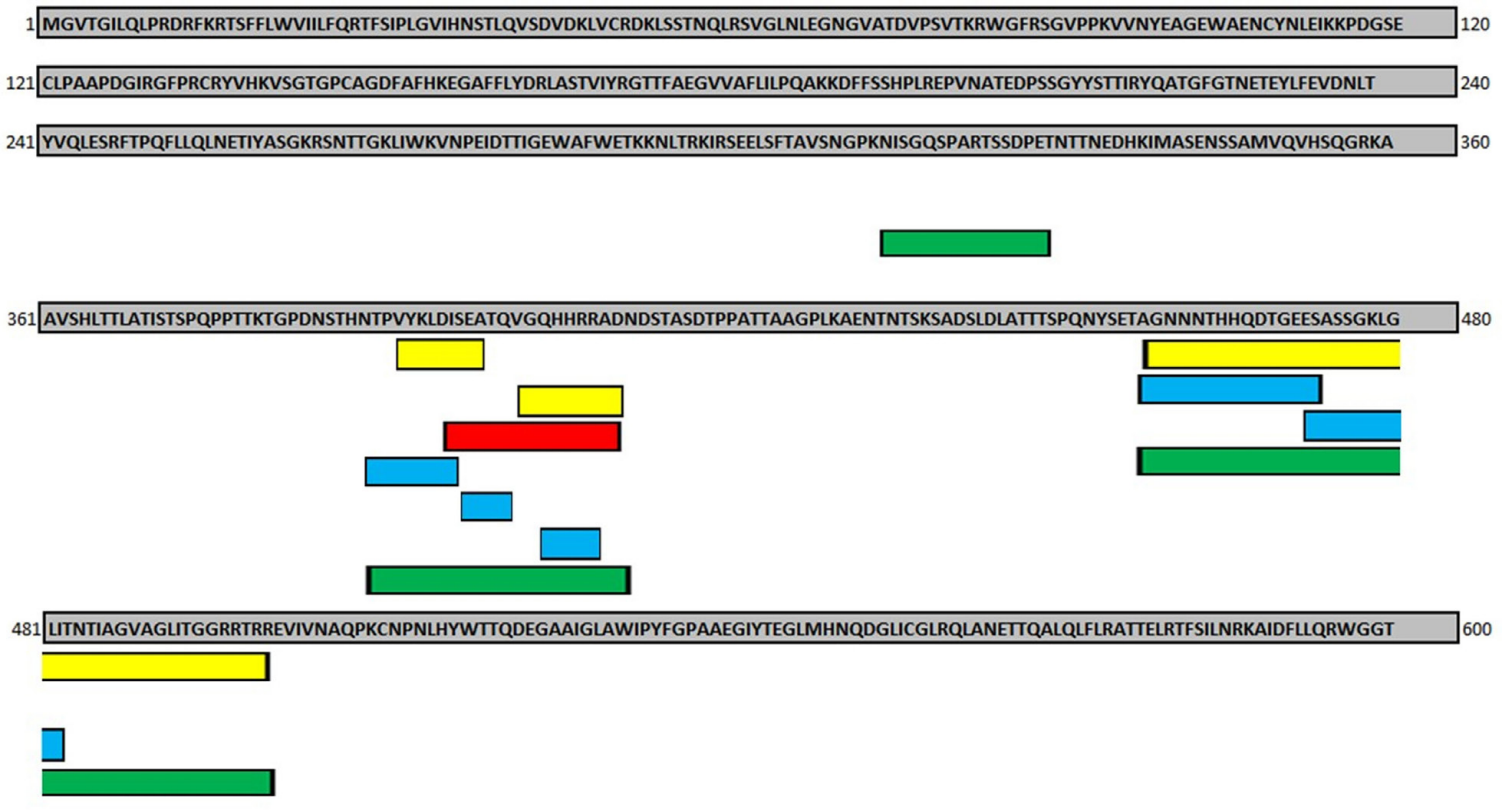
**Results from the *in silico* epitope analysis of the African *Zaire ebolavirus* (ZEBOV) spike glycoprotein sequence (GenBank: AIE11809)**. Regions in red indicate the epitopes reported by Becquart et al. (71), based on reactivity with sera collected from human survivors as an indication of the induction of neutralizing humoral responses. Regions in yellow indicate the epitopes reported by Vaughan et al. (72) as EBOV-related B-cell epitopes found in the Immune Epitope Database. Regions in blue indicate conserved regions of ZEBOV for the African continent overlapping with the epitopes reported in both articles. Regions in green indicate matches of the conserved regions found in the bioinformatics analysis and the epitopes reported in the aforementioned articles.

The key steps involved in the development of plant-made vaccine prototypes include the following: design putative functional immunogens and develop genetically engineered plants expressing the antigen or establishing viral vector-based platforms for transient expression, estimate yields and antigenic properties of the target antigen, assess the immunogenic potential of the candidate vaccine in test animals in terms of protective immunity and safety, and perform clinical trials once preclinical studies have provided acceptable outcomes (Figure 2).

## Possibilities for the Design of Immunogens

A successful proof of concept on EBOV plant-based vaccines will include the design and production of full-length viral proteins such as GP, matrix viral protein (VP40), and NP antigens, as well as chimeric proteins carrying conserved protective epitopes capable of inducing anti-EBOV neutralizing antibodies. Examples of the latter approach include the following linear epitopes: EQHHRRTDN, VIKLDISEA, and LITNTIAGV (25). Hopefully, the current knowledge on the protective EBOV sequences as well as the technologies to produce heterologous proteins in plant cells will accelerate the development of plant-made vaccine candidates against EBOV.

**Figure 3:**
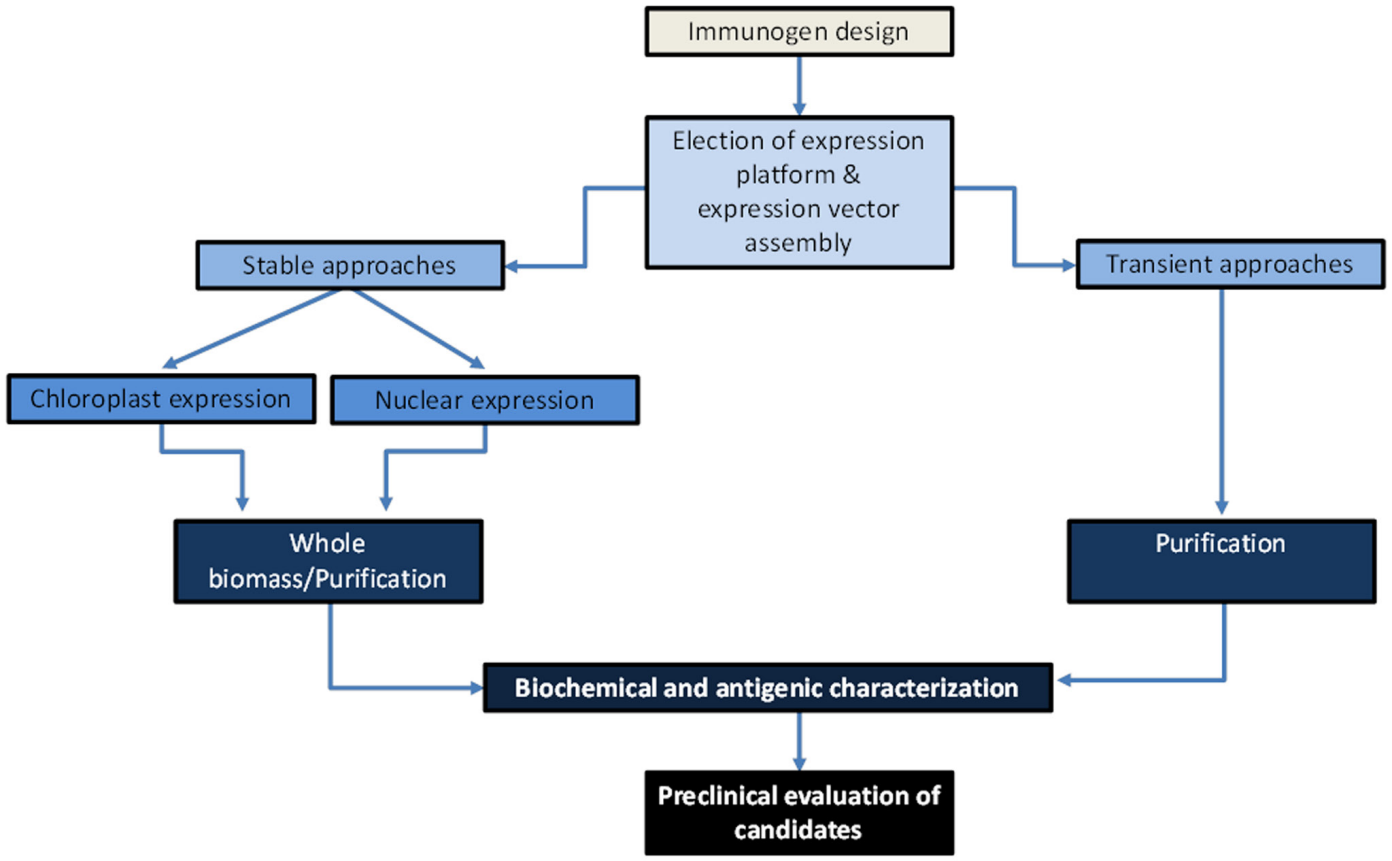
**Scheme on the path for development of *Ebola virus* plant-based vaccine candidates**. Antigens will be designed to serve as strong mucosal immunogens, and coding genes will be assembled into expression vectors elected according to the expression approach to be assessed. Antigen production can be achieved transiently through strategies of chimeric virus (first-generation vectors) or deconstructed virus (second-generation vectors, e.g., agroinfiltration with viral pro-vectors), or stably through a nuclear transformation approach (transformation mediated by *Agrobacterium* or physical methods) or chloroplast transformation approach (transformation mediated by physical methods). A subsequent characterization of the plant-made antigens will comprise estimating antigen yields and antigenic properties. During preclinical trials, it is envisioned that transient approaches will serve as a high productive platform that will render parenteral vaccines after a purification process, which are ideal as prime doses, while stable transformed lines from edible crops may serve as low-cost oral vaccines formulated with freeze-dried plant biomass.

Regarding epitope vaccines, Wilson et al. (25) reported a GP epitope that is conserved among all Ebola viruses demonstrating that a specific mAb was able to protect mice from a lethal EBOV infection. Subsequently, it was found that although some EBOV GP epitopes induce an antibody-dependent enhancement of EBOV infection, antibodies against other specific EBOV GP epitopes were required to control an EBOV infection (73). Another recent study demonstrated that a linkage region (aa 393–556) of the GP (called MFL) contains a furin cleavage site and an internal fusion loop responsible for important viral functions (74). This region was the major contributor to immunogenicity in terms of the induction of humoral immune responses and neutralizing antibodies against the EBOV (75). Interestingly, the study by Becquart et al. (71), using sera from infected patients, identified specific B-cell epitopes in four EBOV proteins [GP, NP, and matrix viral protein (VP40 and VP35)]. Among them, the specific immunodominant VP40 and GP epitopes were detected by IgG antibodies from asymptomatic individuals and symptomatic Gabonese EBOV infected survivors, respectively. These findings strongly suggest that an effective epitopic subunit vaccine should induce humoral IgG responses targeting specific GP and VP40 epitopes. One interesting approach in the design of an epitope-based vaccine capable of triggering protective immune responses is the use of immunoinformatic tools. In this regard, the potential of inducing both humoral and cell-mediated immunity by T and B cells against the EBOV epitopes was recently assessed by Khan et al. (76). From the complete proteomes of EBOVs, the amino acid sequences were retrieved using UniProt Knowledge Base and bioinformatic analyses were conducted to study antigenicity, solvent-accessible regions, surface accessibility, flexibility, MHC class-I-binding epitopes (cellular immune response), and B-cell-binding epitopes (antibody immune response) from those proteins. The *in silico* capability of each protein sequence to initiate an immune response allowed for the identification of the most promissory L protein comprise of 128 amino acids, which is also known as RNA-dependent RNA polymerase. This protein reached the highest antigenicity score in VaxiJen analysis among all the query proteins. The downstream bioinformatic analysis showed that the 9-mer epitope TLASIGTAF was the selected potential epitope-based vaccine candidate for inducing cytotoxic T cell immune responses by considering its overall epitope conservancy (76.60%), human population coverage (53–81%), and the affinity for highest number of MHC-I (HLA) molecules (*n* = 12). Similarly, the L protein was evaluated to identify B cell epitopes and the 9-mer epitope PEEQEQSAE (spanning region from 42 to 50 amino acids) of the L protein was the most potential B cell epitope to induce antibody-mediated immune responses. However, it should be considered that L protein is the last one expressed during viral replication, and thus, a vaccine targeting only this antigen may result in low efficacy. Therefore, vaccine design should contemplate a combination of L protein epitopes with those of early proteins, such as GP. Thus, the combination of experimental data with immunoinformatic prediction approaches opens up a new horizon to design effective multiepitopic vaccines able to induce protective antibody immune responses against the EBOV. In fact, the Immune Epitope Database and Analysis Resource^3^ has reported an integrative immunopredictive and experimental analysis for “functional epitopes.” These epitopes are identified using assays that demonstrate their potential to induce positive outcomes when virus neutralization assays or challenge experiments are performed. A high percentage of the selected epitopes were from the GP (55%) and NP (33%) proteins. The functional EBOV-related B cell epitopes were only found in these two proteins (72). On the other hand, an *in silico* analysis to identify EBOV conserved sequences among the EBOV variants, matching with the abovementioned functional analysis, has allowed the identification of a set of promising GP *Zaire* EBOV B-cell epitopes comprising the following sequence: NISGQSPARTSSDPE, NTPVYKLDISEATQVGQHHRRAD, and TAGNNNTHHQDTGEE SASSGKLGLITNTI AGVAGLITGGRRTR. These sequences are considered promising candidates for multiepitopic vaccine design (Figure 3).

Since vaccines administered through mucosal membranes, mainly by oral route, are the most convenient approach for mass vaccination, the developments in this direction are a priority. However, epitopes are not good immunogens *per se* and thus must be coupled to carrier proteins or adjuvant sequences that favor uptake and efficient antigen presentation. Antigen uptake at the mucosa can be aided by the use of transmucosal carriers, such as the B subunits from either the cholera toxin (CTB) or the enterotoxigenic *E. coli* heat-labile toxin (LTB). These proteins produce oligomeric structures that bind the GM1 ganglioside on the surface of gut epithelial cells, mediating the translocation into the submucosal compartment where the antigen can be processed by dendritic cells with the subsequent induction of adaptive immune responses (77). These properties enable both CTB and LTB to be highly immunogenic and serve as effective carrier proteins and adjuvants for unrelated coupled antigens (78, 79). Therefore, the designed chimeric proteins, through genetic fusion, are proposed as candidates that could result in immunogens capable of inducing strong anti-EBOV antibody responses using oral immunization under the plant-based vaccine concept. This idea is also supported by the proven oral immunogenic activity of CTB- and LTB-based chimeric antigens produced in plants (79, 80). Therefore, specific EBOV epitopes in the form of CTB- or LTB-based chimeras could serve as candidates to induce immunoprotective humoral responses against the EBOV. Other strategies might include the design of chimeras comprising target epitopes and cell penetrating peptides, such as those derived from the HIV-1 Tat protein or the *Drosophila melanogaster* Antennapedia homeodomain (penetratin), which increase the cellular uptake of large molecules (81, 82).

Another attractive possibility in developing plant-based EBOV vaccines could be based on VLPs. It is well established that plants can synthetize structural viral proteins that self assemble into VLPs. These structures are macromolecular complexes that typically are highly immunogenic due to their complexity. Two types of VLPs can be produced: those based only on structural viral proteins and those based on envelope viral proteins associated to a membrane layer from the plant cell. VLPs derived from *bluetongue virus, Norwalk virus, influenza virus, Hepatitis B virus* (HBV) (nucleocapsid antigen), *human papilloma virus*, and *rotavirus* have been successfully assembled in plants (83). These viral proteins have also been engineered to display unrelated epitopes and thus serve, as in the case of CTB and LTB, as immunogenic carriers. This strategy has been successfully applied in a number of cases (80, 84).

Another alternative for immunogen design consists of recombinant immune complexes (RICs). RICs rely on the production of self-polymerized chimeras, whose monomeric form is comprised of the antigen of interest fused to the heavy chain of a mAb against the same antigen of interest. This has been found to be an effective strategy to increase antigen accumulation in transgenic plants enhancing immunogenicity (85).

## Expression and Delivery Possibilities

High antigen yields will constitute a key factor in the flowchart to define the viability of the vaccination approach. In particular when oral vaccine development is pursued, high doses of antigen are typically required. If this aspect is addressed, the ambitious goal of developing oral vaccines will be greatly favored. Oral vaccines constitute the most attractive immunization approach since they offer easier and safer administration as well as the possibility of inducing mucosal and systemic immune responses. Although low expression levels were a limitation in the initial attempts at exploring the viability of plant-based vaccines, it is envisioned that the current optimized expression platforms will allow the production of the targeted antigens at acceptable yields to reach the required level in the plant biomass that could reasonably constitute an oral dose (86).

Each expression modality possesses particular advantages but at the same time imposes some limitations. Therefore, the selection of the expression platform should follow a case-by-case analysis contemplating the nature of the elected antigen, the delivery approach, and the required time response (Table 3). For instance, viral vector-based systems offer high yields, efficient production of complex glycosylated proteins, and the shortest production time among the plant-based platforms. However, since these processes are based in *Nicotiana* species and agroinfiltration, parenteral vaccines can only be produced after an extensive purification process to obtain an antigen free of bacterial compounds and toxic plant metabolites (87). Therefore, VLP-based EBOV vaccines using the GP or VP40 antigens can be ideally produced in viral vector-based platforms as a quick response to epidemics, parenterally immunizing the population at risk. However, it should be considered that these vaccines will not result in low-cost formulations and will require sterile devices and trained personnel for administration. In fact, there is one approved patent covering the production of EBOV VLPs in plants (88). A report by Phoolcharoen et al. (89), where a geminiviral vector was used for expression of the EIC in leaves of *N. benthamiana*, illustrates the potential for producing functional EBOV antigens at convenient yields.

**Table 3:** **Identified expression options for specific EBOV immunogens using the available plant expression technologies**.

		Available expression platforms		
		Stable nuclear transformation	Transient nuclear vector-mediated expression	Chloroplast expression
		Advantages: well established for edible crops to be used in oral vaccines	Advantages: high yields	Advantages: high yields
		Limitations: expression is often low and should be optimized	Limitations: current methodologies require purification due to the use of *Agrobacterium* and non-edible hosts, thus are recommended for parenteral vaccines production	Limitations: protocols available for few edible crops, long time required for transformation
Possible immunogens	Full-length glycoprotein	Highly recommended	Highly recommended	Not recommended due to lack of glycosylation
		Reports related to this approach: a patent registered by D’aoust et al. (88) claims the expression of virus-like particle (VLP) in plants, comprised of the influenza transmembrane domain, and the cytoplasmic tail; fused to ectodomain from a non-influenza virus trimeric surface protein, covering EBOV	Reports related to this approach: a patent registered by D’aoust et al. (88) claims the expression of VLPs in plants, comprised the influenza transmembrane domain, and the cytoplasmic tail; fused to ectodomain from a non-influenza virus trimeric surface protein, covering EBOV	
	Full-length VP40	Highly recommended	Highly recommended	To be determined
	Multi-epitope proteins	Highly recommended	Highly recommended	Highly recommended
	Immune complexes	Highly recommended	Highly recommended	To be determined
			Reports related to this approach: Phoolcharoen et al. (89) expressed EBOV immune complex in leaves of *Nicotiana benthamiana* using a geminiviral vector	

In the case of transplastomic approaches, the average yields are lower than those of the viral expression vector but still considered convenient. However, it should be considered that the time for generating transplastomic lines is very long and no complex posttranslational modifications, such as glycosylation, occur in this organelle (90). Therefore, this platform is ideal for the production of epitope-based vaccines where no complex antigens requiring glycosylation are targeted. One attractive avenue consists in the use of edible plant species for which chloroplast transformation has been established. This is the case of lettuce, which was used for the production of some vaccines (91–93).

By contrast, stable nuclear expression also offers high biosynthetic capacity and propagation of cells in bioreactors. The time for generating transformed lines depends on the species but is generally shorter than that required for transplastomic approaches. Yields are in general modest but can be optimized using several approaches such as organelle targeting and formation of protein bodies (94). Interestingly, several edible plant species can be efficiently transformed. For instance, lettuce can be transformed efficiently using *Agrobacterium tumefaciens* (95). Another interesting species is the carrot (*Daucus carota*), for which there are efficient protocols for *Agrobacterium*-mediated transformation (96, 97). This host, *D. carota*, is relevant considering that the first plant-made biopharmaceutical for human use introduced into the market (Taliglucerase), which is a glucocerebrosidase for Gaucher’s disease treatment, was expressed in carrot cell cultures. This fact implies that the production processes and the regulatory framework are already in place for this system (98). In fact, the company that developed this process is also working on validating the oral delivery of a recombinant product using carrot cells (99).

## Considerations for Plant-Based EBOV Vaccines in Preclinical Evaluations

The antigenic and immunogenic properties of the target immunogens should be evaluated through molecular and immunological analyses. At the same time, these techniques will allow antigen quantification. For strategies based on LTB or CTB as carriers, proper folding and formation of pentameric structures produced in plants can be assessed by evaluating their interaction with the GM1 receptor in GM1–ELISA assays. Positive signals for this analysis imply that the chimeric protein is assembled into the pentameric form, and therefore, a proper uptake can be expected at the mucosa.

On the other hand, VLPs are usually detected *via* electron microscopy, which provides evidence of their successful assembly. VLPs have the ability to stimulate strong immune responses upon oral delivery. In fact, it is considered that the compact and highly ordered structures of VLPs can provide resistance to digestive proteases (100). It is worth mentioning that the antigenic proteins for the HBV are one of the most studied models for production of plant-derived VLPs (101). It has been widely demonstrated that HBV VLP carriers spontaneously assemble in plant cells, resulting in VLPs that preserve their structure (102).

In terms of posttranslational modifications, glycosylation is of particular relevance in the production of vaccines based on GP. It should be considered that distinct glycosylation processes occur in plants with respect to mammalian cells: complex type glycans in plants possess, unlike GPs in mammals, a β(1,2′)-xylose residue, and/or an α(1,3′)-fucose residue linked to the core glycan (103), and a second *N*-acetylglucosamine (GlcNAc) is enzymatically added to the mannose core, and lack of β(1,4′)-galactose- and sialic acid-containing complex type glycans as well as the bi-antennary *N*-glycans production found in mammals. However, these differences on glycosylation do not necessarily result in a non-functional or low quality product. In fact, in the case of vaccines, there is the possibility that differential glycans associated with the plant-derived antigen could enhance immunogenicity (104). Moreover, recent advances in the plant glycoengineering allow human-like glycomodification and optimization of the desired glycan structures for enhancing safety and functionality of recombinant vaccines (105).

Another important consideration is the use of rodent or large animal models mentioned above that will allow assessing the immunogenicity and immunoprotective potential of the plant-made EBOV vaccine candidates.

Two cases can be highlighted as examples of how the above mentioned methodologies have resulted in desirable vaccine prototypes: (i) a plant-based vaccine candidate against malaria has been produced in plants by using a transplastomic expression approach. Fusion proteins consisting of CTB along with the antigens malaria apical membrane antigen-1 (AMA1) or merozoite surface protein-1 were produced in lettuce and tobacco leaves; these candidates induced humoral responses and protective immunity against a cholera toxin challenge. Moreover, both oral and injectable vaccination with CTB-AMA-1 resulted in the blocking of the parasite from entering the erythrocytes (13). It should also be considered that LTB has been successfully produced in several crops, including corn and potato (106, 107). The potato-made LTB was used to conduct a pioneering Phase I clinical trial, showing its capacity to achieve seroconversion with no major adverse effects following an oral immunization scheme (106). (ii) A vaccine prototype against *Mycobacterium tuberculosis* has been developed following an approach based on RICs. The early secreted Ag85B and the latency-associated Acr antigen were expressed in tobacco plants as fusion proteins along with an anti-Acr antibody. Remarkably, Bacillus Calmette–Guérin (BCG)-immunized mice boosted intranasally with TB-RICs showed a significant reduction in *M. tuberculosis* lung infection in comparison with the group immunized only with BCG (108).

Based on the current evidence on the efficacy of several plant-based vaccines orally administered (16), it is proposed that plant-based formulations may result in strong immune responses that could provide immunoprotection against EBOV. It should also be considered that plant-based vaccines could be applied as oral boosters in prime-boost immunization approaches. This focus has proven useful in many plant-based vaccine prototypes, including those against *Yersinia pestis*, HBV, and *M. tuberculosis* (11, 108, 109). All of the aspects mentioned in this article are critical in defining the feasibility of performing evaluations of the plant-based vaccine candidates in clinical trials.

## Concluding Remarks

There is an urgent need to develop efficacious vaccines against the EVD. Although preclinical trials are continuously reported for EBOV vaccine prototypes, efforts to develop low-cost vaccine production platforms should be contemplated. Plant-made vaccines offer the potential to address large-scale vaccine production at low cost, thereby facilitating the success of global vaccination programs, especially in developing and poorer countries where coverage is problematic mainly due to vaccine costs. Only one plant-made vaccine candidate has been developed against the EBOV thus far. Therefore, systematic efforts are required to expand this important research field. The path to address this objective will include (i) the design of protective antigens based on the current knowledge of the EBOV immunogenic determinants and on eficacious immunogenic carriers, preferably those that are highly effective in mucosal membranes; (ii) achieving sufficient antigen yields in edible plant biomass to establish models for oral immunization using minimally processed plant biomass; and (iii) validating the safety as well as the immunogenic and immunoprotective potential of plant-made vaccine candidates in test animals.

Each expression platform offers particular advantages, and the election should be based on the nature of the chosen antigen, the required time response, and desired delivery route. In conclusion, the continuing effort toward the development of plant-made vaccines prototypes could lead to important data to select approaches with the realistic goal of providing efficacious and cost-effective strategies to protect against the EVD. Thus, we encourage research in this direction to accelerate the fight against this deadly disease.

## Author Contributions

SR-M designed the content of the review, wrote the plant sciences aspects, and corrected the full manuscript. RN-G performed the epitope analysis, made the figures and participated in the general writting of the manuscript. CA designed in part the content of the review, and wrote the immunology and virology aspects.

## Conflict of Interest Statement

The authors declare that the research was conducted in the absence of any commercial or financial relationships that could be construed as a potential conflict of interest.

## Footnotes

^1^http://www.who.int/en/.

^2^https://clinicaltrials.gov.

^3^www.iedb.com.
